# Time for a change

**DOI:** 10.7554/eLife.00791

**Published:** 2013-04-30

**Authors:** Sabrina E Sanchez, Marcelo J Yanovsky

**Affiliations:** 1 **Sabrina E Sanchez** is at the Instituto de Investigaciones Bioquímicas de Buenos Aires, Leloir Institute, Buenos Aires, Argentinassanchez@leloir.org.ar; 2 **Marcelo J Yanovsky** is at the Instituto de Investigaciones Bioquímicas de Buenos Aires, Leloir Institute, Buenos Aires, Argentinamyanovsky@leloir.org.ar

**Keywords:** circadian rhythm, transcription factors, evening element, circadian rhythms, Arabidopsis

## Abstract

The circadian clock of *Arabidopsis*, a popular model organism for plants, is more complex than expected, with negative feedback loops based on the repression of gene expression having a less exclusive role than previously thought.

**Related research article** Hsu PY, Devisetty UK, Harmer SL. 2013. Accurate timekeeping is controlled by a cycling activator in *Arabidopsis*. *eLife* **2**:e00473. doi: 10.7554/eLife.00473

**Image** A simplified version of the circadian clock of *Arabidopsis*

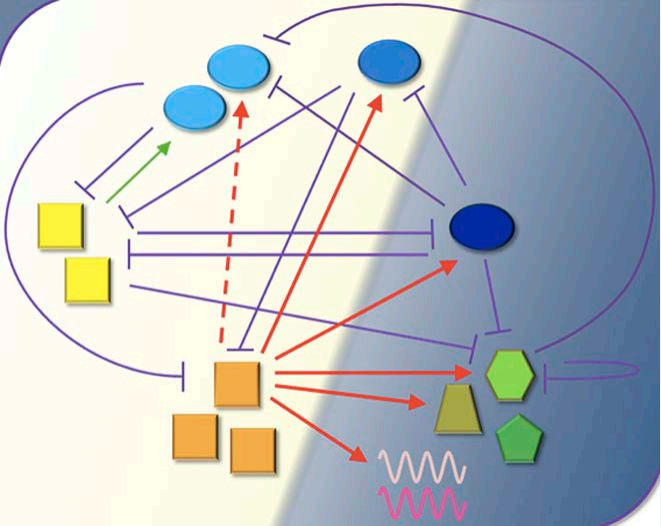

Most organisms have evolved the ability to tell the time, which helps them to cope with daily changes in their environment. Daily rhythms in leaf movements were actually described more than 2000 years ago by Androsthenes, who worked as a scribe for Alexander the Great, and we now know that these circadian rhythms persist with a period of approximately 24 hours, even when the environmental conditions remain constant (McClung, 2006). Indeed, circadian clocks control processes that range from the sleep-wake cycle in humans to the seasonal regulation of flowering time in crop plants (Young and Kay, 2001). A better understanding of the molecular mechanisms underlying these circadian rhythms could lead to important biomedical and agricultural applications.

Our current understanding of the circadian clock in plants is mostly based on transcription factors that mutually repress each other (Gendron et al., 2012; Huang et al., 2012; Pokhilko et al., 2012). In particular, the transcription factors CCA1 and LHY, which are mostly produced in the morning, are thought to repress the expression of the gene that codes for another transcription factor, TOC1, which is mostly produced in the evening and, in turn, represses expression of the genes *CCA1* and *LHY* (see Figure 1). Although this model predicts many properties of real circadian clocks, it is difficult to avoid thinking that processes other than gene repression must also be involved. Now, in *eLife*, Polly Hsu, Upendra Devisetty and Stacey Harmer, all from the University of California Davis, report strong evidence that a protein named RVE8 performs such a positive role: it does this by promoting rather than repressing the expression of certain ‘clock’ genes at certain times of day (namely, in the late afternoon and early evening; Hsu et al., 2013).

**Figure 1.**
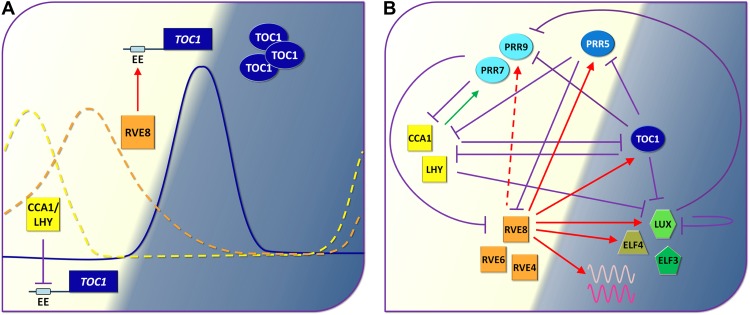
The circadian clock of *Arabidopsis*. (**A**) By demonstrating that a transcription factor called RVE8 increases the expression of hundreds of evening phased genes including the clock gene called *TOC1* in *Arabidopsis*, Hsu, Devisetty and Harmer have shown that the circadian clocks of plants are more complex (see part **B**) than previously thought (Hsu et al., 2013). Early in the morning (left; light background), the proteins CCA1 and LHY repress the expression of *TOC1* by binding to the evening element (EE) in the promoter region of this gene: the suppression is represented by the violet line with the flat end. As the day progresses, however, other proteins down regulate the genes that code for CCA1 and LHY, and this allows the expression of *TOC1* to increase at dusk: the solid blue line shows the level of *TOC1* mRNA. RVE8 contributes to this increase by binding to the evening element of the *TOC1* gene. Levels of *TOC1* mRNA decrease during the evening as RVE8 levels (orange dotted line) fall off and CCA1 protein levels (yellow dotted line) start to rise again. (**B**) Simplified representation of the circadian clock of *Arabidopsis*: again, the violet lines represent a protein repressing the expression of a gene, the green arrow shows that CCA1/LHY increase the production of certain PRR proteins during the day, and the red arrows show the roles played by RVE8 and two similar proteins (RVE4 and RVE6) during the day and in the evening (dark background), as revealed by Hsu et al. The dashed red arrow indicates an interaction that only occurs in specific conditions. The three PRR proteins shown here are similar to TOC1: they are produced throughout the afternoon and early evening and they act to reduce the production of CCA1 and LHY. Finally ELF4, LUX and ELF3 are components of an evening protein complex that represses the expression of morning genes such as *PRR9*. The waves represent evening output genes induced by RVE8.

The origin of this story can be traced back to 2000, when Harmer and co-workers found that a short DNA sequence named the evening element was over-represented in the promoter regions of genes that are mostly expressed at dusk (Harmer et al., 2000). In particular, the evening element is present in the promoter region for *TOC1*, and the expression of this gene is repressed by the transcription factors CCA1 and LHY binding to the evening element (Alabadi et al., 2001). However, two findings suggested that other events contributed to the fact that the expression of *TOC1* peaked in the evening.

First, mutations in the evening element sometimes decrease rather than increase gene expression, which suggests that the evening element can also mediate the binding of transcription factors that activate gene expression (Harmer and Kay, 2005). Second, biochemical experiments revealed that a particular protein complex binds to the evening element in the afternoon in both wild-type plants and in mutant plants that do not produce CCA1 or LHY (Harmer and Kay, 2005). This afternoon-phased DNA binding activity could actually be involved in promoting the expression of early evening genes.

The latest work by Hsu et al. on the circadian clock of *Arabidopsis*—which is widely used as a model plant organism—provides strong evidence that RVE8, a transcription factor that is similar to CCA1 and LHY, regulates genes with peak expression in the early evening. First, the UC Davis team identified hundreds of genes that were either induced (that is, switched on) or repressed (switched off) by RVE8. Moreover, they observed significant differences between these two types of genes: the genes that were induced by RVE8 were those that possess an evening element in their promoter region and are mostly expressed in the early evening under daily light/dark cycles, whereas those that were repressed by RVE8 are mostly expressed in the morning.

The UC Davis team also provides convincing evidence that RVE8 directly acts to induce evening genes, while morning genes were regulated indirectly by this protein. This conclusion is consistent with previous work which showed that RVE8 binds the evening element both in vitro and in planta, and that *rve8* mutants display alterations to their circadian rhythms (Rawat et al., 2011; Fariñas and Mas, 2011). However, the importance of RVE8 to the circadian clock was not fully understood. First, the circadian period of *rve8* mutants was only one hour longer than that of wild-type plants. Second, the afternoon-phased DNA binding activity described above was also detected in the *rve8* mutants. Third, transcriptomic analysis revealed that only a very small subset of genes were potential targets for RVE8 (Hsu and Harmer, 2012).

Hsu et al. suggest that the modest effect of *rve8* mutations on the circadian clock of *Arabidopsis* is because two similar proteins—RVE4 and RVE6—are also involved. Indeed, the circadian period of triple *rve4;rve6;rve8* mutants is four hours longer than that of wild-type plants. Moreover, these triple mutants do not display the afternoon-phased DNA binding activity observed in wild-type plants. Finally, in the triple mutants the expression of the afternoon/early evening-phased genes was altered much more than that of the morning-phased genes. All these observations strongly support the conclusion that RVE8, along with RVE4 and RVE6, all play a key role in the circadian clock of *Arabidopsis* by switching on the afternoon/early evening genes, which then go on to control the expression of morning genes, thus starting the circadian cycle again (Figure 1).

The work of Hsu, Devisetty and Harmer makes it clear that the circadian clocks of plants are more complex than previously thought, and that new models are needed to understand how all these interactions lead to 24 hour rhythms. Ultimately this improved understanding could have practical applications: for example, it might become possible to make specific crop plants flower at the most appropriate time of the year in different geographic locations.
